# A Review of Nitrogen-Doped Graphene Aerogel in Electromagnetic Wave Absorption

**DOI:** 10.3390/mi14091762

**Published:** 2023-09-12

**Authors:** Ze Wu, Xinke Yao, Youqiang Xing

**Affiliations:** School of Mechanical Engineering, Southeast University, Nanjing 211189, China

**Keywords:** graphene aerogel, nitrogen atom doping, electromagnetic waves absorption

## Abstract

Graphene aerogels (GAs) possess a remarkable capability to absorb electromagnetic waves (EMWs) due to their favorable dielectric characteristics and unique porous structure. Nevertheless, the introduction of nitrogen atoms into graphene aerogels can result in improved impedance matching. In recent years, nitrogen-doped graphene aerogels (NGAs) have emerged as promising materials, particularly when combined with magnetic metals, magnetic oxides, carbon nanotubes, and polymers, forming innovative composite systems with excellent multi-functional and broadband absorption properties. This paper provides a comprehensive summary of the synthesis methods and the EMW absorption mechanism of NGAs, along with an overview of the absorption properties of nitrogen-doped graphene-based aerogels. Furthermore, this study sheds light on the potential challenges that NGAs may encounter. By highlighting the substantial contribution of NGAs in the field of EMW absorption, this study aims to facilitate the innovative development of NGAs toward achieving broadband absorption, lightweight characteristics, and multifunctionality.

## 1. Introduction

In the advent of the “5G” era, the integration of various electronic devices into our society has brought about unparalleled convenience in both our work and daily lives. However, this rapid evolution and dominance of electronic equipment in the market has also raised a pressing concern: EMW pollution. Prolonged exposure to electromagnetic radiation can have severe repercussions on the human nervous system, potentially leading to irreversible damage, including the development of cancer. Furthermore, electromagnetic interference poses a significant risk, as it can cause malfunction or failure in electronic equipment [1,2].

In the military sector, radar technology plays a critical role in detecting and locating targets by emitting EMWs and analyzing their reflections. However, the accuracy of radar detection can be significantly compromised by EMW pollution and interference. Therefore, the development of radar stealth technology is crucial in bolstering national military strength. The key to achieving radar stealth lies in reducing the radar cross section (RCS) [3,4]. This can be achieved by altering the shape of military equipment or applying absorbing materials to the surface to attenuate the energy of EMWs. However, attaining the desired stealth effect typically requires a combination of methods that integrate appearance stealth with the reduction in EMW energy to minimize the RCS.

To mitigate the impact of EMW pollution on daily life and related industries, the development of EMW absorbing materials is essential. Absorbing materials can be classified into two categories based on their mechanisms for attenuating EMWs: dielectric loss and magnetic loss types [4]. Dielectric loss is influenced by two major factors: polarization relaxation and conduction loss. Polarization relaxation, which does not lead to reverse radiation, is considered an ideal form of dielectric loss. It encompasses interfacial polarization caused by electronegativity differences at different interfaces and dipole polarization resulting from crystal defects. These polarization mechanisms within the material help with absorbing and dissipating the energy of the EMW.

In contrast, magnetic loss mechanisms in absorbing materials involve natural resonance at low frequencies, eddy current loss, and exchange resonance at extremely high frequencies. Natural resonance is commonly found in ferrite materials and occurs due to the interaction between the internal constant magnetic field and the external changing magnetic field. This resonance phenomenon is closely related to the intensity of the magnetic field. Eddy current loss, on the other hand, happens when induced eddy currents in magnetic materials dissipate energy in response to an external changing magnetic field. The intensity of this loss depends on the permeability and conductivity of the material.

In the pursuit of creating an effective absorbing material, it is crucial to adhere to two fundamental principles [3,5]. Firstly, the material should exhibit minimal reflection when EMWs encounter its surface. This is achieved through impedance matching characteristics, where the material’s properties are carefully designed to match those of the surrounding medium, thereby minimizing the reflection of EMWs. Secondly, the material should maximize the absorption of EMWs within its internal structure, adhering to the principle of maximum attenuation. This involves meticulously designing the material’s composition and structure to efficiently absorb and dissipate the energy of the EMWs. Developing absorbing materials that possess both impedance matching and attenuation capabilities represents a challenging yet indispensable endeavor in the field of radar stealth technology.

Traditional absorbing materials, such as carbon materials, metals, oxides, conductive polymers, and metal sulfides, usually rely on a single loss mechanism, which poses challenges in achieving the desired absorption effect. To overcome this limitation, the development of magnetic/dielectric composite materials has emerged as an effective approach for achieving efficient EMW absorption. By integrating both magnetic and dielectric loss mechanisms, these composite materials can significantly enhance their absorption capabilities and deliver superior overall performance in absorbing EMWs.

Graphene and reduced graphene oxide aerogels (GA and RGAs) have garnered considerable interest in recent years due to their porous structure and unique three-dimensional conductive network [6]. These features help to reduce the agglomeration of graphene sheets, leading to multiple scattering, conduction loss, and polarization loss at the interface of the EMW when they enter the material. This allows the aerogels to effectively attenuate and absorb the EMW. Additionally, the high compressibility, electrical conductivity, thermal conductivity, and low density of GA make them suitable for various applications, including supercapacitors, energy storage, sensors, and EMW absorption.

For example, Wang and co-workers prepared graphene aerogels with excellent mechanical compressibility and flame retardancy [7]. These aerogels exhibited a minimum reflection loss (RL_min_) of up to −61.09 dB and an effective absorption bandwidth (EAB, RL < −10 dB) of 6.30 GHz. Furthermore, GAs can be used as frameworks to support or connect other functional materials, such as magnetic ferrites, metals, and polymers, to enhance their EMW absorption efficiency. For instance, Jiang and co-workers prepared a composite aerogel using a freeze-drying process with graphene as a framework and silicon carbide whiskers as a coating, achieving an RL_min_ of −47.3 dB at 10.52 GHz [8]. Additionally, the combination of polyaniline and graphene significantly improved the EMW absorption capacity of pure GA, with an RL_min_ of −42.3 dB at a matching thickness of 3 mm [9].

Apart from graphene materials, Ti_3_C_2_T_x_ MXene is also widely used for EMW absorption due to its excellent physical and chemical properties. MXene materials promote the conduction loss and polarization loss of EMWs entering the material [5,10,11]. However, single graphene/MXene-based aerogels may not achieve optimal absorption effects due to weak impedance matching. To overcome this, they are often combined with dielectric heterogeneous materials to improve their dielectric properties [5,10]. For example, Liang and co-workers introduced one-dimensional magnetic Ni nanochains into graphene/MXene-based aerogels, achieving an RL_min_ of −75.2 dB and an EAB of 7.3 GHz [12]. Peng and co-workers prepared MnO_2_/Ti_3_C_2_T_x_/RGO composite aerogels, and the RL_min_ reached −66.5 dB when the thickness was only 2.0 mm [13].

To enhance the absorption performance of GA, one commonly used technique is heteroatom doping, with nitrogen doping being particularly popular and extensively studied. Heteroatom doping increases the dielectric loss and polarization loss of the aerogels, ultimately promoting an impedance-matching balance [1,14]. The most commonly used nitrogen dopants include ethylenediamine (EDA), ethylenediaminetetraacetic acid (EDTA), urea, and other nitrogen sources, which can introduce nitrogen atoms into the graphene structure. Nitrogen is a preferred doping atom for several reasons. Firstly, nitrogen atoms have comparable molecular weight and atomic size to carbon atoms, which ensures that the introduction of nitrogen does not significantly disrupt the overall structure of GA. Secondly, nitrogen is relatively electronegative, meaning it attracts electrons towards itself and contributes electrons to the conjugated system of GA. This promotes conduction loss, which is crucial for efficient EMW absorption. Finally, the addition of nitrogen atoms introduces numerous intrinsic defects in the graphene carbon structure, disrupting the hexagonal lattice and enhancing polarization loss [15].

The NGA exhibits excellent electrochemical performance and energy storage characteristics, making it a preferred material for the fabrication of supercapacitors [16,17,18,19] and electrode materials [20,21,22,23,24,25,26]. Moreover, its chemical reactivity allows it to act as a catalyst [27,28,29] and photocatalyst for oxygen reduction reactions [30,31,32,33,34,35]. The porous structure of the NGA also grants it outstanding adsorption properties, making it suitable for water pollution treatment or degrading organic matter [36,37,38,39,40,41,42]. Additionally, it is utilized in the production of biosensors [43,44,45].

Nevertheless, one of the most notable applications of NGAs lay in the field of EMW absorption. At present, NGAs are a hot topic in three-dimensional structural materials that are combined with magnetic ferrites, carbon nanotubes, and other nanoparticles [46,47,48,49,50]. By examining the various studies and advancements in this area, this paper seeks to present a comprehensive overview of the capabilities and potential future directions of NGAs as EMW absorbers.

## 2. Synthesis of Nitrogen-Doped Graphene Aerogel

Nitrogen can be incorporated into carbon through three main methods: in situ doping (or pre-doping), post-doping, and direct pyrolysis [14].

In the in situ doping method, nitrogen is introduced during the growth of carbon materials, typically through vapor deposition techniques like chemical vapor deposition (CVD). Ammonia (NH_3_) is commonly used as the nitrogen dopant in this method. During the CVD process, nitrogen atoms are incorporated into the carbon lattice structure, resulting in nitrogen-doped carbon materials.

The post-doping method involves introducing nitrogen atoms into the carbon material simultaneously with the reduction of graphene oxide (GO). This method typically involves the use of nitrogen-containing compounds or functional groups, which react with the oxygen-containing groups on the GO during the reduction process. Therefore, it is widely used in the preparation of NGAs due to its ability to introduce vacancy defects, which promote polarization loss of EMWs within the material.

The direct pyrolysis method involves the direct pyrolysis of nitrogen-containing precursors, such as melamine or nitrogen-containing polymers. These precursors are subjected to high temperatures, causing them to decompose and release nitrogen.

Each of these doping methods offers different advantages and can be used to tailor the properties of nitrogen-doped carbon materials based on the specific application requirements.

**Figure 1:**
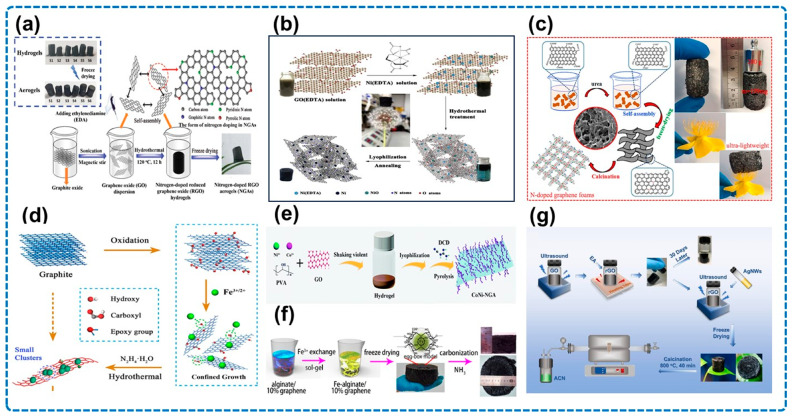
Different selections of nitrogen dopants and schematic illustrations of aerogel samples. (**a**) EDA, NGAs. Reproduced with permission from ref. [51], Copyright (2020) Wiley-VCH GmbH. (**b**) EDTA, N-rGA/Ni. Reproduced with permission from ref. [52], Copyright (2019) Elsevier. (**c**) urea, N-doped graphene foams. Reproduced with permission from ref. [53], Copyright (2019) Elsevier. (**d**) N_2_H_4_•H_2_O, Fe_3_O_4_-NG. Reproduced with permission from ref. [54], Copyright (2017) Elsevier. (**e**) DCD, CoNi-NGA. Reproduced with permission from ref. [55], Copyright (2020) The Royal Society of Chemistry. (**f**) NH_3_, Fe_2_N@C-NPs/N-GAs. Reproduced with permission from ref. [56], Copyright (2016) American Chemical Society. (**g**) ACN, AgNWs@NGAs. Reproduced with permission from ref. [57], Copyright (2022) Elsevier.

Most NGAs are prepared by the post-doping method, and Figure 1 shows different selections of nitrogen dopants during the synthesis of aerogel samples.

In Figure 1a, EDA was chosen as a nitrogen dopant to inject nitrogen atoms into the graphene crystal structure and also a reducing agent for GO in the synthesis of the NGA, which was assisted by the hydrothermal self-assembly and freeze-drying processes [51].

Figure 1b illustrates that EDTA worked not only as a nitrogen doping agent but also the chelating agent of metal ions, which can be combined with the hydrothermal method and calcination process to prepare the composite where nickel nanoparticles are anchored on the three-dimensional NGA (N-rGA/Ni) [52].

As is shown in Figure 1c, urea acted as a nitrogen dopant in the preparation of NG foam, and the sample had a high porosity and an open network structure through the hydrothermal self-assembly and freeze-drying processes [53].

Figure 1d portrays the Wang and co-workers’ method of choosing hydrazine (N_2_H_4_•H_2_O) as a nitrogen dopant to incorporate nitrogen into the Fe_3_O_4_-NG, and the reduction of GO and the self-assembly of the final sample were realized by the hydrothermal method [54].

In Figure 1e, dicyandiamide (DCD) functioned as a nitrogen dopant in the preparation of CoNi-NGA, and it was placed in an inert gas atmosphere to introduce nitrogen atoms into the material [55].

Figure 1f illustrates the Liu and co-workers’ method of using an NH_3_ atmosphere as the nitrogen dopant to prepare Fe_2_N@C-NPs/N-GAs, along with the methods of freeze-drying and carbonization [56].

As is shown in Figure 1g, acetonitrile (ACN) was the crucial factor for the incorporation of nitrogen, which was mixed with samples in a vacuum tube furnace to synthesize AgNWs@NGAs [57].

EDA, EDTA, and urea are commonly used as dopants for nitrogen doping in graphene materials due to their simplicity in the synthesis process and their ability to introduce stable nitrogen-doping effects. These dopants can be easily incorporated into the graphene structure during synthesis, allowing for the controlled introduction of nitrogen-doped atoms by adjusting the amount of dopant added. The presence of nitrogen dopants in graphene materials promotes the generation of vacancy defects and the formation of carbon–nitrogen bonds. These defects and bonds contribute to the modification of the electronic structure and enhance the dielectric properties of the materials.

For the synthesis of NGAs, the solvothermal self-assembly process and freeze-drying are commonly employed techniques. The solvothermal conditions, such as temperature, pressure, and reaction time, can affect the nitrogen-doping effect and the formation of GAs [58]. Optimizing the solvothermal conditions is crucial for achieving the desired nitrogen-doping levels and the desired porous structure of the aerogels.

## 3. N-Doped Graphene Aerogel for Electromagnetic Wave Absorption

### 3.1. Pure N-Doped Graphene Aerogel for Electromagnetic Wave Absorption

Existing studies have indeed identified three main types of nitrogen incorporation in NG systems: graphitic nitrogen, pyrrolic nitrogen, and pyridinic nitrogen. The bonding state of N atoms in NGAs can be effectively regulated by selecting appropriate nitrogen dopants. Regardless of the source of nitrogen, the EMW absorption performance of NGAs is directly related to the concentration of these three N-bonds. Therefore, it is necessary to take the nitrogen doping dose as a variable to fully consider its influence on the doping effect in graphene aerogels in practical studies.

Graphitic nitrogen primarily contributes to conduction loss within the material, enhancing its electrical conductivity. It facilitates the movement of electrons through the graphene lattice, allowing for efficient conduction of electricity. On the other hand, pyrrolic and pyridinic nitrogen primarily promote dipole relaxation loss [14,59]. These nitrogen configurations disturb the uniform charge distribution on the graphite surface, and they introduce polar groups within the graphene structure, leading to increased dipolar interactions with EMWs [60]. This promotes the dissipation of energy through the dipole relaxation processes, thus enhancing absorption properties.

**Figure 2:**
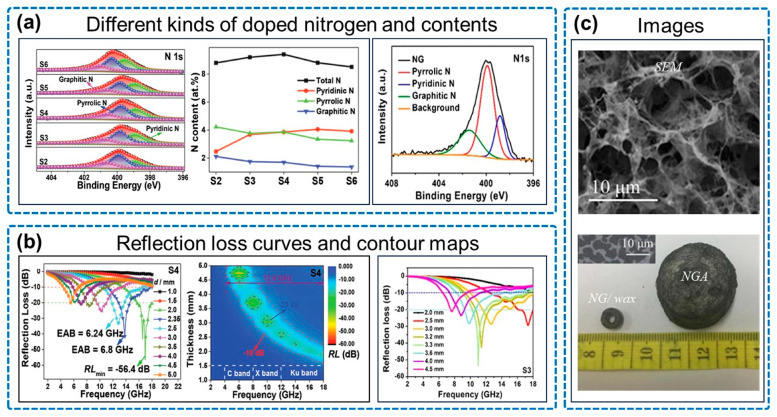
EMW absorption properties of pure NGA. (**a**) Different kinds of doped nitrogen and contents. (**b**) Reflection loss curves and contour maps. (**c**) Images. Reproduced with permission from ref. [51], Copyright (2020) Wiley-VCH GmbH; reproduced with permission from ref. [61], Copyright (2017) Springer Science+Business Media, LLC, part of Springer Nature.

The studies of pure NGAs provide insights into the respective contributions of various nitrogen types to EMW absorption (Figure 2a). Shu and colleagues demonstrated that the nitrogen doping content directly influenced the EM parameters and absorption capacity of the aerogel [51]. Among the nitrogen types, pyridinium nitrogen was responsible for conduction loss, while pyrrole nitrogen contributed to dipole polarization. By controlling the nitrogen doping dose, it was found that the best absorption performance was achieved at a nitrogen content of 9.41 at%, resulting in an RL_min_ of −56.4 dB and an EAB of 6.8 GHz, covering the entire Ku band (Figure 2b).

Similarly, experiments conducted by Zhou and colleagues confirmed that pyrrole nitrogen was the primary nitrogen doping type responsible for polarization loss in the system (Figure 2a). The addition of nitrogen atoms disrupted the ideal sp^2^ hybridization of carbon atoms in the graphene structure, leading to the formation of sp^3^ defects and disordered sites [61]. This resulted in defective polarization relaxation and electron dipole polarization relaxation, thereby promoting the dielectric loss in graphene. The best absorption effect was achieved at a filling amount of 3.6 wt%, with an RL_min_ of −53.25 dB and an EAB of 8.15 GHz (Figure 2b).

**Figure 3:**
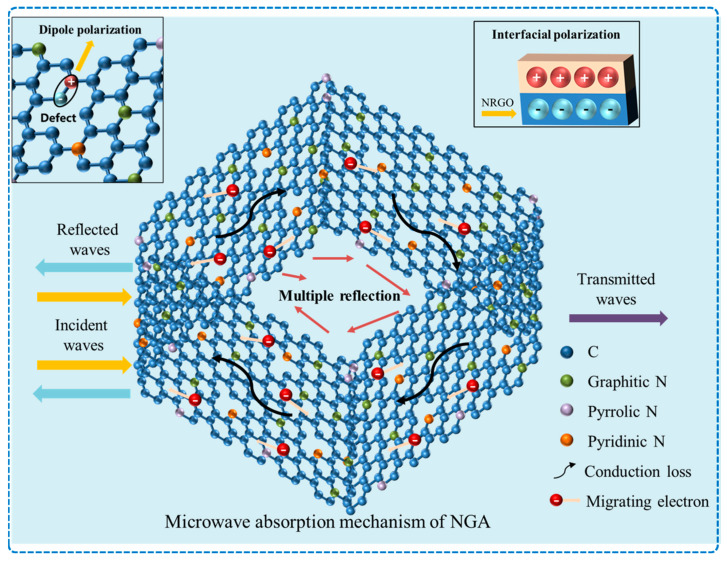
Electromagnetic wave absorption mechanism of NGA.

In terms of an EMW absorption mechanism, pure NGAs possess the three-dimensional conductive structure of GAs and exhibit unique EMW absorption abilities due to the nitrogen doping process. Based on a comprehensive literature research, three main loss modes have been identified (as shown in Figure 3):

(1) Conduction loss: The three-dimensional conductive network formed by the GA provides a pathway for electron transport. The introduction of nitrogen atoms adds extra electrons to the system, thereby increasing the conduction loss in the graphene network. Additionally, under the influence of alternating EM fields, according to Cao’s electron jump model [62], electrons can absorb energy from the EMWs and move through the graphene network by migration and jumping, thus leading to conduction loss.
(2) Polarization loss: Defects and oxygen-containing functional groups present at the edges of graphene layers, such as hydroxyl and carboxylic groups, act as polarization centers, generating defect polarization and dipole polarization. Furthermore, the incorporation of nitrogen atoms disrupts the sp^2^ hybridization of the graphene lattice, leading to the formation of sp^3^ defects and disordered sites [14]. This results in the generation of dipole polarization with C–N electric dipoles as centers, as well as defect polarization with nitrogen atoms as centers. Additionally, the presence of heterogeneous interfaces between the graphene and paraffin matrices can result in interface polarization [63].
(3) Multiple scattering: The three-dimensional network structure of the aerogel and layered structure of graphene allows for multiple reflections and scatterings of EMWs within the material. This phenomenon results in the attenuation of EMW energy.

Similar to GAs, using a single component alone may not achieve the ideal EMW absorption effect. Therefore, it is necessary to compound NGAs with the addition of magnetic materials or other dielectric heterogeneous materials to enhance the EMW absorption performance of the material.

### 3.2. N-Doped Graphene Aerogel/Magnetic Metal for Electromagnetic Wave Absorption

At a theoretical level, the introduction of metal ions can indeed enhance both the real and imaginary components of the dielectric constant, as well as the dielectric loss coefficient of the materials. Zhang and co-workers have demonstrated that reduced GO aerogels prepared using a cation-assisted strategy exhibited improved EMW absorption [64]. This can be attributed to the ability of cations to attract negative charges, thereby improving the aggregation of graphene sheets. Moreover, the presence of metal ions can induce increased polarization loss, thereby facilitating the absorption of EMWs.

Nickel ions (Ni^2+^) and cobalt ions (Co^2+^) are commonly used metal ions to enhance the absorption of EMWs. Magnetic particles originating from these ions contribute to the magnetic loss component, which plays a crucial role in EMW absorption. The presence of these magnetic particles can cause additional dissipation of EMW energy, leading to improved absorption performance.

**Figure 4:**
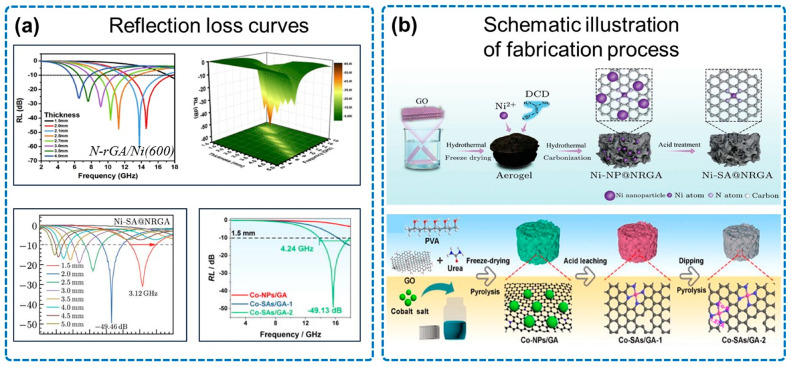
Properties of NGA/magnetic metal nanoparticles. (**a**) Reflection loss curves, including N-rGA/Ni, Ni-SA@NRGA, and Co-NPs/GA, Co-SAs/GAs. (**b**) Schematic illustration of fabrication process, including Ni-SA@NRGA and Co-SAs/GAs. Reproduced with permission from ref. [52], Copyright (2019) Elsevier; reproduced with permission from ref. [65], Copyright (2022) Chinese Physical Society and IOP Publishing Ltd.; reproduced with permission from ref. [66], Copyright (2022) AIP Publishing.

Figure 4 presents the properties of the NGA/magnetic metal nanoparticles composite. According to Figure 4b, Suo and co-workers prepared Ni-SA@NRGA material by using a hydrothermal method and carbonization process [65], while Xu and co-workers prepared an ultra-light Co-SAs/GAs by using secondary ion adsorption and defect anchoring strategies [66]. In Xu’s experiment, the graphene network was etched using sulfuric acid to create abundant mesoporous sites as defect sites for the secondary adsorption of cobalt ions. This promotes dipole polarization loss and defect polarization loss in the material. Figure 4a illustrates the reflection loss curves, the N-rGA/Ni(600) sample prepared by Tang and co-workers obtained an RL_min_ of −60.8 dB at 13.7 GHz and an EAB of 5.1 GHz [52], and the Ni-SA@NRGA reached an RL_min_ of −49.46 dB. In addition, the Co-SAs/GAs obtained an RL_min_ of −49.13 dB at 13.7 GHz and an EAB of 4.24 GHz, with a matching thickness of merely 1.5 mm.

The detailed preparation methods of metal-NGAs will be further summarized in the following section.

To further enhance the EMW absorption capabilities of aerogels, researchers have explored different nanoparticle structures, in addition to the anchoring method mentioned earlier. These studies have investigated various nanoparticle shapes, including cubes, hollow structures, linear structures, and flower-shaped structures. These shapes facilitate multiple scattering of microwave materials, thereby ensuring the maximum absorption of EMW energy. Among these shapes, the cube is one of the structures commonly introduced into the system. For example, Gao and co-workers reported RGO aerogels loaded with hollow Co_1-x_Ni_x_O nanocubes [67], with an ultra-broad EAB of 9.5 GHz and an RL_min_ of −53.3 dB.

**Figure 5:**
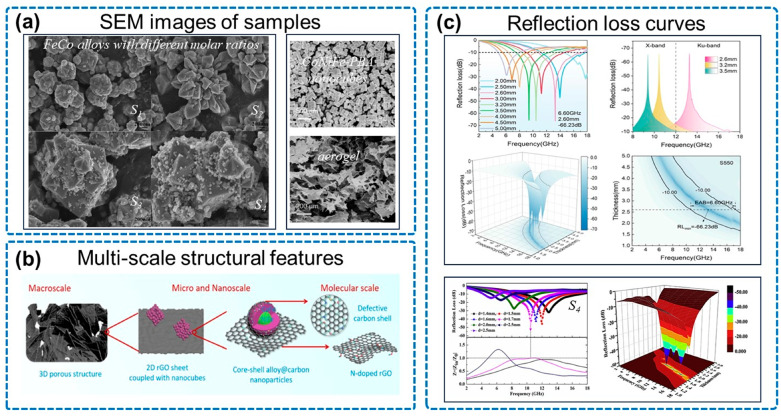
Properties of NGA/magnetic metal nanocubes. (**a**) SEM images of samples, including FeCo alloys with different molar ratios (S_1_, S_2_, S_3_, S_4_), CoNiFe-PBA nanocubes and CoNiFe-PBA/GO aerogel derivative. (**b**) Multi-scale structural features of CoNiFe-PBA/GO aerogel derivatives. (**c**) Reflection loss curves, including S_550_ of CoNiFe-PBA/GO aerogel derivatives and S_4_ of FeCo alloys. Reproduced with permission from ref. [68], Copyright (2019) Elsevier; reproduced with permission from ref. [71], Copyright (2022) Elsevier.

Meanwhile, cobalt-nickel-iron cubes (polyhedra) are widely used due to their excellent dielectric properties. As is shown in Figure 5, the polyhedral FeCo alloy nanoparticles synthesized by Zhou and co-workers exhibited an RL_min_ of −52.20 dB at 10.48 GHz, with a sample thickness of only 1.7 mm [68]. The NiFe-MOF/N-rGO prepared by Zhao reached an RL_min_ of −72.28 dB at 10.82 GHz, and the EAB was up to 7.14 GHz (9.74–16.88 GHz) [69]. Additionally, the FeCo cubes can also be combined with other components to further enhance absorbing properties, such as the lightweight Fe–Co/NC/rGO composite reported by Wang and co-workers, which had a broad EAB of 9.12 GHz (8.88–18 GHz) [70]. Wei and co-workers synthesized an NGA anchored by magnetic CoNiFe nanocubes [71], with an RL_min_ of −66.23 dB and an EAB of 6.6 GHz at a thickness of 2.6 mm when the filling content was only 1.1 wt%. Figure 5b illustrates that the CoNiFe nanocubes formed a large number of heterogeneous interfaces with the graphene sheets, intensifying interface polarization, and the NiFe and CoFe alloy cores constituting the CoNiFe nanocubes were responsible for magnetic losses, including natural resonance and eddy current losses [72].

**Figure 6:**
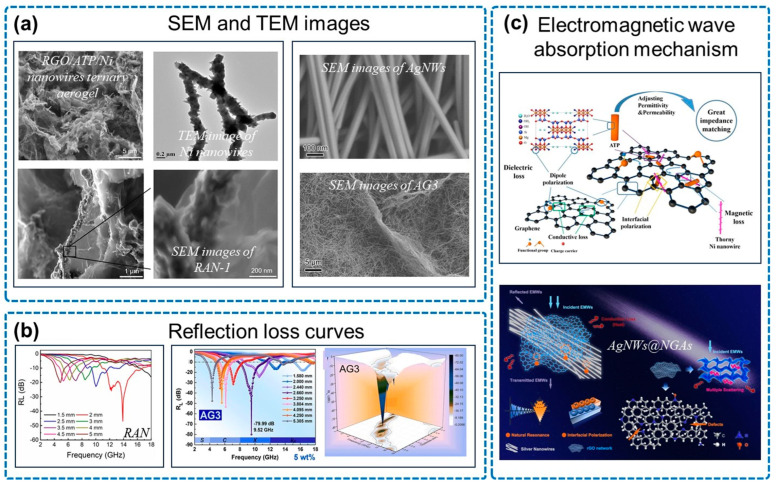
Linear structures in NGA/magnetic metal composites. (**a**) SEM and TEM images, including SEM images of RGO/ATP/Ni nanowires ternary aerogel(RAN-1), TEM image of Ni nanowires and SEM images of AgNWs and AgNWs@NGAs(AG3). (**b**) Reflection loss curves, including RAN and AG3. (**c**) EMW absorption mechanism. Reproduced with permission from ref. [57], Copyright (2022) Elsevier; reproduced with permission from ref. [73], Copyright (2021) Elsevier.

Linear structures, as illustrated in Figure 6, are commonly utilized to enhance the absorbing properties of materials. Sun and co-workers reported the use of barbed nickel nanowires to enhance the magnetic losses in GAs [73], with an RL_min_ of −47 dB and an EAB of 6.8 GHz. The AgNWs@NGAs designed by Shu and co-workers reached an RL_min_ of −79.99 dB at 9.52 GHz, and the EAB was up to 3.5 GHz [57]. As is shown in Figure 6c, the introduction of linear structures facilitates magnetic losses, encompassing natural resonance, exchange resonance, and eddy current losses.

### 3.3. N-Doped Graphene Aerogel/Magnetic Oxide for Electromagnetic Wave Absorption

In addition to the synergistic interaction between NGAs and magnetic metal nanoparticles, magnetic oxides can be utilized to enhance the dielectric properties and matching properties of NGAs. Many studies have investigated the application of various ferrites, including pure ferrite, cobalt ferrite, and nickel ferrite, as potential materials for EMW absorption.

(1) Pure ferrite

The combination of pure ferrite and NGAs is commonly used in catalysts for batteries or chemical oxygen reduction reactions. Studies have shown that composites of ferrite or single iron atoms with graphene exhibit good dielectric properties [74,75,76]. Furthermore, the combination of ferrite and NG enhances wave-absorbing properties. For example, Li and co-workers prepared N-GN/Fe_3_O_4_ hybrid materials using an in situ solvothermal method and achieved an RL_min_ of −65.3 dB [77]. Wang and co-workers prepared an Fe_3_O_4_ cluster absorbent nitrogen-doped graphene using a growth restriction strategy, with an RL_min_ of −53.6 dB [54].

(2) Cobalt and nickel ferrite

**Figure 7:**
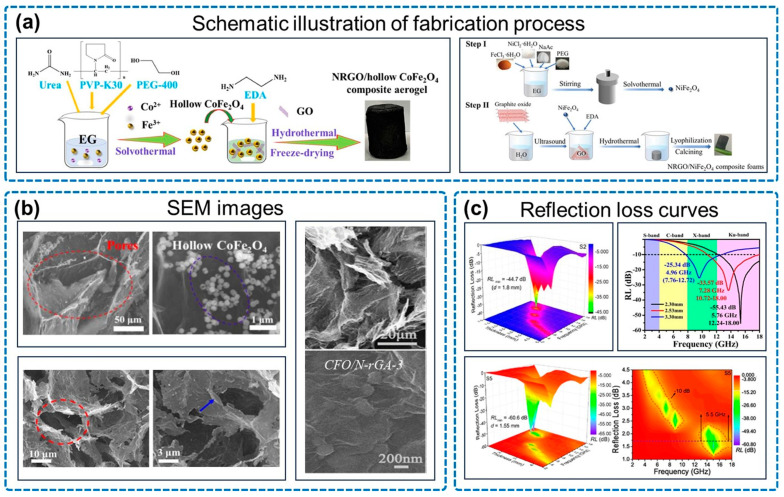
Properties of NGA/magnetic oxide. (**a**) Schematic illustration of fabrication process, including NRGO/hollow CoFe_2_O_4_ composite aerogels and NRGO/NiFe_2_O_4_ composite foams. (**b**) SEM images, including NRGO/hollow CoFe_2_O_4_ composite aerogels (S_2_), NRGO/NiFe_2_O_4_ composite foams (S_5_), and CFO/N-rGA-3. (**c**) Reflection loss curves, CoFe_2_O_4_ composite aerogels (S_2_), CFO/N-rGA-3 and NRGO/NiFe_2_O_4_ composite foams (S_5_). Reproduced with permission from ref. [79], Copyright (2022) Elsevier; reproduced with permission from ref. [80], Copyright (2022) Elsevier; reproduced with permission from ref. [81], Copyright (2021) Elsevier.

Cobalt ferrite and nickel ferrite are widely used in combination with nitrogen-doped graphene due to their excellent EMW absorption ability. For example, Wang and co-workers prepared NGAs embedded with CoFe_2_O_4_ nanoparticles and achieved an RL_min_ of −60.4 dB and an EAB of 6.48 GHz at a mass ratio of 1:2 GO to CoFe_2_O_4_ [78]. Deng and co-workers reported an NRGO/NiFe_2_O_4_ prepared by a solvothermal–hydrothermal two-step method (Figure 7a), achieving an RL_min_ of −60.6 dB and an EAB of 5.5 GHz [79].

Various studies have also explored innovative approaches with cobalt ferrite nanoparticles. As is shown in Figure 7a, Xu and co-workers prepared NRGO/hollow CoFe_2_O_4_ composite aerogels, achieving an RL_min_ of −44.7 dB and an EAB of 5.2 GHz [80]. Wang and co-workers prepared a raspberry CoFe_2_O_4_ (CFO) cluster decorated NRGO aerogel, achieving an RL_min_ of −55.43 dB and promoting multiple reflections of EMWs within the material [81].

Further research directions include exploring innovative particle structures and considering improving composite material performance by anchoring NGAs with cobalt ferrite or nickel ferrite nanoparticles.

(3) Composite ferrite

In some cases, the use of single ferrites may not achieve the desired absorption effect. Therefore, researchers have explored the preparation of composite ferrites by combining metal solutions of different components, such as cobalt, nickel, and zinc metal ions.

**Figure 8:**
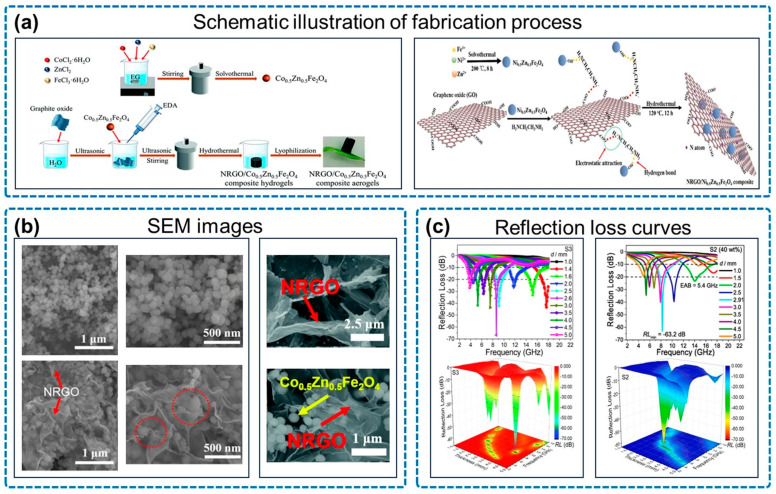
Properties of NGA/composite ferrite. (**a**) Schematic illustration of fabrication process, including NRGO/Co_0.5_Zn_0.5_Fe_2_O_4_ composite aerogels and NRGO/Ni_0.5_Zn_0.5_Fe_2_O_4_ composite. (**b**) SEM images, including NRGO/Ni_0.5_Zn_0.5_Fe_2_O_4_ composite (S_2_), and NRGO/Co_0.5_Zn_0.5_Fe_2_O_4_ composite aerogels (S_3_). (**c**) Reflection loss curves, including NRGO/Co_0.5_Zn_0.5_Fe_2_O_4_ composite aerogels (S_3_) and NRGO/Ni_0.5_Zn_0.5_Fe_2_O_4_ composite (S_2_). Reproduced with permission from ref. [82], Copyright (2021) The Royal Society of Chemistry; reproduced with permission from ref. [83], Copyright (2019) Elsevier.

As is shown in Figure 8, Shu and colleagues synthesized an NRGO/Co_0.5_Zn_0.5_Fe_2_O_4_ composite aerogel by using the solvothermal and hydrothermal self-assembly methods [82], and the RL_min_ reached −66.8 dB with an ultra-thin thickness of only 1.6 mm. They also synthesized an NRGO/Ni_0.5_Zn_0.5_Fe_2_O_4_ composite using GO as a template, achieving an RL_min_ of −63.2 dB and an EAB of 5.4 GHz (12.0–17.4 GHz) [83].

The introduction of composite ferrites into an NGA requires careful control of two key factors: nitrogen doping dosage and the ratio between different elements. These factors play a crucial role in determining the absorption properties of the composite material.

(4) Other oxides

Fu and co-workers designed a novel LaFeO_3_-decorated NGA (LFO/N-rGO) by the hydrothermal method [84], and when the mass ratio of the LaFeO_3_ nanoparticles:GO:urea is 1:5:7.5, LFO/N-rGO achieved an RL_min_ of −64.5 dB at 9.2 GHz, and the EAB was up to 6.72 GHz.

In addition to the magnetic oxides mentioned above, rare earth oxides, such as cerium oxide (CeO_2_), also possess unique dielectric properties. Liu and colleagues reported that a “matryoshka doll”-like cerium oxide microsphere exhibited highly efficient EMW absorption, with an RL_min_ of −71.3 dB at 14.5 GHz [85]. Huang and colleagues prepared a cerium oxide/porous carbon composite using pine cones, which achieved an RL_min_ of −56.04 dB [86]. Gao and colleagues proposed a two-dimensional carbon-based nanocomposite loaded with nitrogen-doped cerium oxide, and at a filler content of 10 wt%, the RL_min_ reached −58.24 dB [87]. The excellent absorption performance of cerium oxide is attributed to the vacancy defects it causes in the material system, which promote defect polarization and interface polarization due to the heterogeneous interface between cerium oxide and the main framework.

From a technological perspective, the microwave absorption performance of composite materials containing cerium oxide and the main framework can vary. For example, Zhao and colleagues prepared a cerium oxide/nitrogen-doped carbon fiber composite through the electrospinning method, achieving an RL_min_ of −42.59 dB and an EAB of 8.48 GHz (at 2.5 mm) with a 3 wt% filling amount of polyvinylidene fluoride (PVDF) [88]. Wu and colleagues prepared a nitrogen-doped reduced GO/cerium oxide (NRGO/CeO_2_) composite aerogel using a hydrothermal method, with the RL_min_ of −50.0 dB at an ultra-thin thickness of 1.9 mm, and the EAB reached 5.7 GHz (12.3–18.0 GHz) [89].

(5) Combination with sulfides

The graphene aerogel compound with sulfide has proved to have excellent absorption properties. For example, Wu and co-workers prepared CuS@rGO composite aerogels, with an RL_min_ of −55.1 dB, and the EAB could reach up to 8.44 GHz [90]. Among various sulfides, molybdenum disulfide (MoS_2_) has the ability to induce relaxation phenomena [91]. Consequently, it is frequently utilized in conjunction with graphene [92].

In summary, magnetic oxides offer numerous possibilities to enhance the microwave absorption performance of NGAs. Moreover, their synergistic effects with other substances can lead to significant improvements.

### 3.4. N-Doped Graphene Aerogel/Carbon Nanotubes for Electromagnetic Wave Absorption

The combination of NGAs and carbon nanotubes has been demonstrated to enhance the EMW absorption properties of these materials [93]. Zhao and colleagues demonstrated that graphene aerogels decorated with one-dimensional CoNi chains and carbon nanotubes exhibited excellent absorption properties, with an RL_min_ of −50.8 dB at a thickness of 1.8 mm and an EAB of 8.5 GHz (18–26.5 GHz) covering the whole K band [94]. Interestingly, during the investigation of NGAs and carbon nanotube composites, it was discovered that the carbon nanotubes are not found in isolation; instead, they are typically accompanied by the formation of metal nanoparticles. This implies that the composites are heterogeneous multi-component media.

For example, Xu and colleagues synthesized an NRGO aerogel containing pod-like nitrogen-doped carbon nanotubes and FeNi nanoparticles [95]. Even at a filling volume of only 10 wt%, the RL_min_ reached −39.39 dB. This phenomenon is not limited to aerogel experiments. Zhang and colleagues grew CoNi nanoparticles in nitrogen-doped carbon nanotube arrays on ultra-thin reduced GO sheets (3D CoNi/NGCT) [96]. Zhao and colleagues synthesized a CoNi/reduced GO aerogel using solvothermal and carbonization methods [97]. Zhang and colleagues prepared a reduced GO (rGO)/nitrogen-doped carbon (N-C)/FeNi compound with an RL_min_ of −68.87 dB at 12.08 GHz [98].

Xu and colleagues explained the reason for this phenomenon in the process of preparing nitrogen-doped carbon nanotubes/reduced GO aerogels [55]. Under an electron microscope, it was observed that the content of carbon nanotubes produced by choosing a solution containing different metal ions varied, which directly affected the EMW absorption properties. This is because the reduced metal nanoparticles, such as Ni, Co, and Fe, act as catalysts for catalyzing the growth of carbon nanotubes, and for doping nitrogen into carbon nanotubes and into reduced GO. Among the metals, cobalt has the best catalytic effect. During the experiment, polyvinyl alcohol (PVA) was selected as the crosslinking agent between GO and metal ions. After freeze-drying, the sample was mixed with DCD in an inert gas atmosphere to obtain the final sample. This strategy facilitates the preparation of mono-metallic (Fe, Co, and Ni) and bimetallic (CoNi and CoFe) based aerogels, which can be extended to other types of metal-based aerogels.

**Figure 9:**
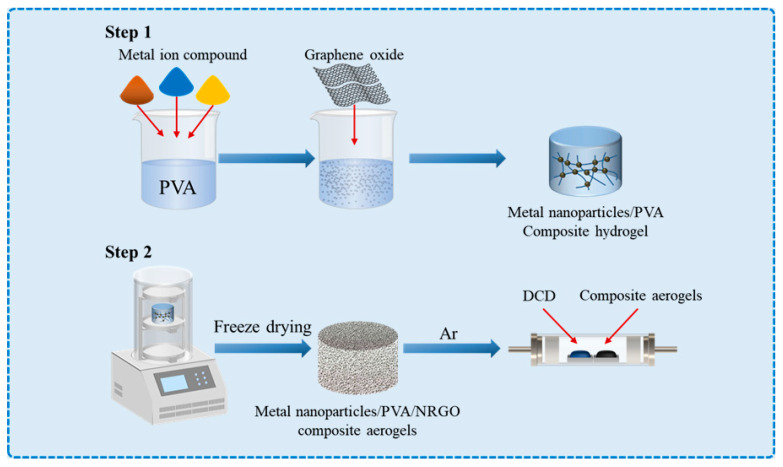
General schematic construction processes of Metal nanoparticles/NRGO composite aerogels.

As is shown in Figure 9, the general methods for preparing mono-metal/bimetallic NGA are summarized as follows:

Firstly, select the corresponding specific solution containing metal ions, disperse it into a PVA solution, and add the GO aqueous solution to prepare the corresponding hydrogel.

In the second step, the hydrogel was freeze-dried to obtain the aerogel containing PVA, GO, and the corresponding metal salts. Then, DCD and the aerogel were heated in proportion and treated in an inert gas atmosphere to produce the final metal nanoparticles/NRGO composite aerogels.

The incorporation of metal nanoparticles and carbon nanotubes into graphene aerogels can indeed increase the heterogeneous interface between graphene sheets, leading to improved interface polarization and enhanced wave absorption properties. The introduction of multi-walled carbon nanotubes (MWCNTs), in particular, can further promote interfacial polarization and enhance the absorption performance. For instance, Shu and co-workers fabricated a composite foam material consisting of nitrogen-doped reduced GO and multi-walled carbon nanotubes [99], with an RL_min_ of −69.6 dB and an EAB of 4.3 GHz (13.2–17.5 GHz). Similarly, Wan and co-workers prepared an NRGO/MWCNT composite aerogel by hydrothermal self-assembly and freeze-drying two-step method [100], achieving an RL_min_ of −46.3 dB and an EAB of up to 4.2 GHz.

The above studies also show that better microwave absorption properties can be obtained by combining carbon nanotubes with nanoparticles. For example, Ren and co-workers proposed a composite aerogel of carbon nanotubes and graphene nanosheets combined with cobalt ferrite [101]. Qin and co-workers also prepared a graphene aerogel composed of carbon nanotubes and Fe_3_O_4_ nanoparticles [102], with an RL_min_ of −49 dB. Therefore, the absorption properties of carbon nanotubes/nitrogen-doped graphene composite aerogels can be further improved by exploring innovative ways to combine them with other substances.

### 3.5. N-Doped Graphene Aerogel/Polymer for Electromagnetic Wave Absorption

Aerogels composed of graphene and polymer composites have demonstrated remarkable EMW absorption capabilities [103]. For instance, Liu and co-workers successfully synthesized a graphene/polypyrrole aerogel using a straightforward one-step reduction self-assembly process [104], with an RL_min_ of −51.12 dB at 6.4 GHz, and an EAB of 5.88 GHz (10.48–16.36 GHz). Similarly, Zhou and co-workers developed ANF/rGO/PI composite aerogels through freeze-drying and annealing techniques [105], with an RL_min_ of −41.0 dB.

Furthermore, the combination of polymers and graphene aerogels has also shown excellent electromagnetic shielding properties [106,107]. Therefore, it is worth exploring methods to integrate nitrogen-doped aerogels with polymers and other substances, as this approach holds great potential for further enhancing EMW absorption capabilities.

Table 1 compares the EMW absorption properties of representative samples and reveals that a pure NGA may not achieve the desired absorption effect. In the actual synthesis of an NGA, researchers need to consider combining it with other components to improve impedance matching.

## 4. Prospects

(1) Low frequency

In existing studies, the EMW absorption frequency bands of NGAs mostly cover the X-band (8–12 GHz) and Ku band (12–18 GHz). However, they usually do not exhibit the ideal EMW absorption effect at the relatively low-frequency range of 1–8 GHz. Therefore, enhancing the absorption effect of materials in the low-frequency band is a crucial aspect for future research.

In terms of absorption of low-frequency EMWs, Yang and co-workers prepared a new FeCoNi carbon fiber (FeCoNi/CF) and one-dimensional magnetic FeCoNi alloy structure by using an improved electrospinning process [108], with an EAB of 1.3 GHz in a low-frequency electromagnetic field with an ultra-thin thickness of 2 mm. Another notable study by Wu and his team involved incorporating MoO_2_ nanoparticles between porous carbon shells and RGO, which successfully achieved excellent low-frequency absorption performance without the need for magnetic components [109]. Undeniably, these groundbreaking discoveries hold significant implications for the development of materials with exceptional wave-absorption properties.

(2) Multifunctional

In order to cater to the various requirements of different environments, the future focus of wave-absorbing materials is on attaining multifunctionality. However, effectively incorporating multiple functions into a single material still poses a significant challenge. For example, Li and co-workers synthesized a multifunctional aerogel composed of multi-dimensional organic and inorganic components, with an RL_min_ of −59.85 dB at a thickness of 1.5 mm [110]. In another study, Cheng and his team prepared RGO hybrid aerogels with infrared stealth and heat insulation properties [111], achieving an impressive RL_min_ of −63.52 dB and an EAB of 8.45 GHz. Furthermore, the binary aerogel designed by Gao and co-workers was both structurally robust and processable, as well as super-hydrophobic, thermal and freeze-resistant, and the EAB was up to 8.4 GHz [112]. These examples highlight the significant strides that have been made in incorporating multiple functionalities into absorbing materials.

(3) Combination with advanced preparation methods

In the reviewed studies, the majority utilized hydrothermal self-assembly followed by freeze-drying to fabricate aerogels. The freeze-casting method is well-suited for obtaining the desired porous structure and facilitating multiple EMW losses [113]. However, the hydrothermal method necessitates high-temperature and high-pressure conditions, leading to significant energy consumption. In addition, the CVD method is also widely used due to its exceptional controllability for preparing graphene aerogels with diverse morphological structures, but this method requires sophisticated equipment to assist it.

Therefore, it is crucial to investigate advanced methods for the fabrication of graphene aerogels. For instance, Li and co-workers successfully prepared hollow graphene aerogel spheres through a combination of coaxial electrospinning, freeze-drying, and calcination processes [114]. This unique approach resulted in an RL_min_ of −52.7 dB and an EAB of 7.0 GHz. Similarly, Zhi and co-workers employed coaxial electrospinning to fabricate Carbon@RGO/Fe_3_O_4_ aerogel microspheres [115], which exhibited an impressive RL_min_ of −61 dB. These examples demonstrate the possibility of utilizing different process paths to prepare NGAs with desirable EMW absorption properties.

(4) Innovation in structure

The achievement of outstanding wave absorption properties depends heavily on the structural aspects, which can be attained through innovative advancements in both the aerogel itself and its composite components. Undoubtedly, optimizing the structure of the aerogel is a pivotal factor in attaining exceptional wave absorption performance. For instance, Huang and co-workers developed a graphene aerogel with a cellular structure [116]. Remarkably, even at a filling ratio as low as 0.74 wt%, this aerogel demonstrated an RL_min_ of −61.63 dB and an EAB of 7.8 GHz. This example showcases the significant impact of the aerogel structure on wave absorption capabilities.

Another avenue for innovation is the exploration of different component compositions to enhance polarization loss in materials by increasing the presence of heterogeneous interfaces. Zhang and co-workers developed three-dimensional reduced GO/γ-GY (RGO/GY) heterostructures, which exhibited an RL_min_ of −71.73 dB at 10.48 GHz and an EAB of 7.36 GHz [117]. This remarkable performance can be attributed to the increased heterogeneous interfaces within the material.

Similarly, Yang and co-workers designed a multi-dimensional NiCo/C/CNT/rGO aerogel using a MOF derivative [118]. This aerogel, characterized by its rich heterogeneous interface, achieved an impressive RL_min_ of −58.8 dB and an EAB of 7.6 GHz. In another study, Huang and co-workers reported a method of directly using MOF crystals to synthesize MOF/reduced GO aerogels, and the sample could achieve an EAB of 7.92 GHz with the filling content of merely 0.6 wt% [119]. These examples further demonstrate the potential of incorporating diverse components to enhance wave absorption properties by promoting polarization loss through increased heterogeneous interfaces.

In conclusion, future research on NGAs should prioritize several key areas for improvement. Firstly, significant attention should be given to enhancing absorption performance in the low-frequency range as it is crucial for numerous practical applications. Secondly, there is a need to develop multifunctional materials that not only absorb EMWs but also possess additional desirable properties such as mechanical strength or thermal conductivity. Thirdly, exploring different processing methods can provide valuable insights into optimizing the fabrication process and improving the overall performance of the aerogels. Lastly, innovation in the structure of the aerogels, such as designing novel architectures or incorporating other materials, can further enhance their wave absorption capabilities. By addressing these aspects, NGAs can be effectively optimized for various applications that require efficient EMW absorption.

## Figures and Tables

**Table 1:** The comparison of EMW absorption properties of typical samples.

Type	Sample	RL_min_ (dB)	EAB (GHz)	Thickness (mm)
Pure GA	GA [7]	−61.09	6.30	4.81
Pure NGA	NGA [51]	−56.4	6.8	2.0
NGA/magnetic metal	NGA/Ni [52]	−60.8	5.1	2.1
	AgNWs@NGA [57]	−79.99	3.5	2.66
	CoNiFe-PBA/NGA [68]	−66.23	6.6	2.6
NGA/magnetic oxide	CFO/NGA [78]	−60.4	6.48	2.1
	Co_0.5_Zn_0.5_Fe_2_O_4_/NGA [82]	−66.8	5.0	2.6
	CeO_2_/NGA [89]	−50.0	5.7	4.0
NGA/CNT	MWCNTs/NGA [100]	−46.3	4.2	1.4

## Data Availability

Data will be made available on request.
